# A nanochitosan-D-galactose formulation increases the accumulation of primaquine in the liver

**DOI:** 10.1128/aac.00915-23

**Published:** 2024-03-22

**Authors:** F. Gomes, A. C. Ribeiro, G. S. Sanches, H. S. Borges, L. A. U. Takahashi, C. T. Daniel-Ribeiro, A. C. Tedesco, J. W. L. Nascimento, L. J. M. Carvalho

**Affiliations:** 1 Laboratory of Malaria Research, Oswaldo Cruz Institute (IOC/Fiocruz), Reference Center for Malaria Research, Diagnosis and Training, Rio de Janeiro, Brazil; 2 Department of Pharmacology (LaFaCE) - ICB, Federal University of Juiz de Fora (UFJF), Juiz de Fora, Minas Gerais, Brazil; 3 Department of Chemistry, Center of Nanotechnology and Tissue Engineering - Photobiology and Photomedicine Research Group, Faculty of Philosophy, Sciences and Letters, University of São Paulo, Ribeirão Preto, São Paulo, Brazil; The Children's Hospital of Philadelphia, Philadelphia, Pennsylvania, USA

**Keywords:** malaria, primaquine, nanostructure, nanochitosan, liver delivery, G6PD deficiency

## Abstract

Primaquine is the mainstream antimalarial drug to prevent *Plasmodium vivax* relapses. However, this drug can induce hemolysis in patients with glucose-6-phosphate dehydrogenase deficiency. Nanostructure formulations of primaquine loaded with D-galactose were used as a strategy to target the drug to the liver and decrease the hemolytic risks. Nanoemulsion (NE-Pq) and nanochitosan (NQ-Pq) formulations of primaquine diphosphate containing D-galactose were prepared and characterized by their physicochemistry properties. Pharmacokinetic and biodistribution studies were conducted using Swiss Webster mice. A single dose of 10 mg/kg of each nanoformulation or free primaquine solution was administered by gavage to the animals, which were killed at 0.5, 1, 2, 4, 8, and 24 hours. Blood samples and tissues were collected, processed, and analyzed by high-performance liquid chromatography. The nanoformulation showed sizes around 200 nm (NE-Pq) and 400 nm (NQ-Pq) and physicochemical stability for over 30 days. Free primaquine solution achieved higher primaquine Cmax in the liver than NE-Pq or NQ-Pq at 0.5 hours. However, the half-life and mean residence time (MRT) of primaquine in the liver were three times higher with the NQ-Pq formulation than with free primaquine, and the volume distribution was four times higher. Conversely, primaquine’s half-life, MRT, and volume distribution in the plasma were lower for NQ-Pq than for free primaquine. NE-Pq, on the other hand, accumulated more in the lungs but not in the liver. Galactose-coated primaquine nanochitosan formulation showed increased drug targeting to the liver compared to free primaquine and may represent a promising strategy for a more efficient and safer radical cure for vivax malaria.

## INTRODUCTION

Malaria is a significant public health problem worldwide, with an estimated 247 million cases and 619,000 deaths from its complications in 2021. The World Health Organization (WHO) African region accounts for about 95% of the cases and 96% of the deaths globally (1). In the Americas, Venezuela, Brazil, and Colombia accounted for 79% of all malaria cases, predominantly caused by *Plasmodium vivax* (83% of all malaria cases in Brazil), in 2021 (1, 2).

*P. viva*x is the most challenging species when considering malaria elimination due to rapid gametocytogenesis, which facilitates transmission, and to relapses imposed by dormant forms, the hypnozoites. Most available blood schizonticides (e.g., chloroquine) do not destroy these forms. Even if the individual is treated and cured of blood forms, the hypnozoites will not be affected and will eventually “awaken,” leading to a new malaria episode. The only drugs currently effective against hypnozoites belong to the class of 8-aminoquinolines, primaquine and tafenoquine (3, 4). Primaquine has been for many years the only approved drug for a radical cure, that is, the elimination of liver-stage hypnozoites in *P. vivax* and *Plasmodium ovale* infections (5, 6). However, primaquine is usually prescribed to be taken once daily for 14 days, leading to efficacy issues related to poor treatment adherence (7). In addition, the anti-hypnozoite action of primaquine depends on active metabolites arising via cytochrome P-450 isozyme 2D6 (CYP2D6) metabolism, and CYP2D6 polymorphisms may result in the drug’s low efficacy (8, 9). Finally, 8-aminoquinolines may induce hemolysis in patients with glucose-6-phosphate dehydrogenase (G6PD) deficiency, the most common human genetic abnormality, with a higher incidence in a number of malaria-endemic countries (10). G6PD is the first enzyme of the pentose phosphate pathway, the rate-limiting step to produce the reducing factor nicotinamide adenine dinucleotide phosphate (NADPH) needed in cellular antioxidant response (11); therefore, red blood cells (RBCs) from G6PD deficient patients are more vulnerable to pro-oxidant challenges. The oxidative stress caused to RBCs by 8-aminoquinolines in G6PD-deficient patients can cause hemolytic anemia and lead to renal failure and death.

Several strategies have been studied to minimize hemolytic toxicity while maintaining 8-aminoquinoline activity (12), which, in the case of primaquine, requires multiple doses in a cumulative effect. Therefore, decreasing the accumulation of primaquine and its metabolites and their availability to cause damage in RBCs is a potential strategy to prevent hemotoxicity. One rationale is to increase liver uptake of the drug, as the hypnozoites are hosted in hepatocytes. Specific organ uptake can be achieved by encapsulating the desired drug in nanoparticles or conjugating the drug to a carrier recognized by receptors highly expressed in the target cells but not in other cell types. In the case of hepatocytes, the use of ligands binding to receptors such as the asialoglycoprotein receptor, which recognizes carbohydrates (mainly galactose and N-acetylgalactosamine) and exists in the order of 10^5^ per hepatocyte, has been extensively researched for specific drug delivery to the liver (13, 14). This strategy has been employed for several drugs, such as doxorubicin, paclitaxel, and primaquine itself (13, 15). Early attempts of primaquine delivery to the liver using liposomes or aminoacid derivatives achieved success, leading to increased accumulation of the drug in the liver and decreased lethality, allowing the administration of higher doses with resulting higher efficacy in preventing infection in mice inoculated with *Plasmodium berghei* sporozoites (16). Liposomal encapsulation also resulted in slower elimination of primaquine in the urine (17). More recent efforts also showed promise. For instance, a chylomicron nanoemulsion formulation of primaquine resulted in increased uptake of the drug (47% recovery) in the liver 1 hour after intravenous administration in mice, compared to free primaquine (20%) (18). Also, primaquine encapsulated in galactose-coated dendrimers increased drug circulation and liver accumulation (19). Alternative formulation approaches included a synthetic trivalent glyco-ligand (TriGalNAc) conjugate containing primaquine, which resulted in marked accumulation of the drug in the liver of mice and in increased uptake by rat hepatocytes (20) or, interestingly, hybrid primaquine glyco-conjugates that showed improved ability for radical cure of *Plasmodium cynomolgi* infections in rhesus monkeys (21).

Given the promising results achieved in these studies, the use of combined approaches for improved drug delivery to the liver, that is, nanoparticle formulations containing ligands for hepatocyte receptors, arises as a potentially effective means of further boosting liver delivery of primaquine. In the present study, two nanoconstructs, a nanoemulsion and a nanochitosan containing galactose residues and loaded with primaquine, were generated, and their pharmacokinetics and biodistribution properties were evaluated in mice. Since the liver expresses high levels of sugar receptors, we expected that galactose-containing nanoparticles would increase primaquine accumulation in this organ.

## MATERIALS AND METHODS

### Nanostructure preparation and characterization

The nanoemulsion is an oil-in-water emulsion obtained by a spontaneous emulsification process. First, the organic phase with solvent (acetone, J.T. Baker, 99%) was prepared using natural soy phospholipids (Epikuron 75) and oil (Capric/Caprylic Acid Triglyceride Oil, Croda Pharma) at 55°C. This organic solution was then added slowly with magnetic control stirring into the aqueous phase containing the anionic surfactant Pluronic F68 (Sigma-Aldrich), D-galactose (Sigma-Aldrich), and primaquine diphosphate (Sigma-Aldrich). Next, primaquine diphosphate and D-galactose were adjusted to final concentrations of 2.15 mg/mL. The final mixture was allowed under controlled magnetical stirring for 30 minutes. Finally, the organic solvent was removed by evaporation under reduced pressure at 55°C in a rota-evaporator system. Next, the empty nanoemulsion used as a control was prepared similarly to the NE-Pq description above in the absence of primaquine diphosphate and D-galactose.

Chitosan nanoparticles were prepared by the ionic gelation method through the cross-linking of chitosan with tripolyphosphate (TPP) in a ratio of 5:2. For the empty nanoparticle, at room temperature, the neutral solution of TPP (1 mg/mL, Sigma-Aldrich) was added to the acid solution (pH 4.5) of chitosan (2 mg/mL, low molecular weight, Sigma-Aldrich). To prepare the loaded chitosan nanoparticles (NQ-Pq), primaquine diphosphate and D-galactose at the final concentration of 2.22 mg/mL were added to the TPP and left with magnetic stirring for 3 hours under temperature control. An analogous procedure prepared the empty control nanochitosan without the actives. A scheme of the nanostructure preparation is shown in Fig. S1.

The particle size (hydrodynamic diameter) and polydispersity index (PDI) were estimated by dynamic light scattering from a ZetaSizer Nanoseries (Malvern Instruments, Malvern, United Kingdom). This technology used a HeNe (633 nm) laser and a ZEN3600 digital correlator with an angle of θ equal to 173° to the incident beam at 25°C. For sample analysis, the nanoemulsion was dispersed in milli-Q water at a ratio of 1:100. The zeta potential was measured by electrophoretic mobility with the same equipment. The incorporation of activity was obtained with the centrifugation method in a Microcon YM-100 type filter (Millipore, Ireland), at 10,000 × *g* for 1 hour, at 4°C in a Centrifuge 5415 R centrifuge (Eppendorf, USA).

### Animals

Swiss Webster mice (male and female), 8–10 weeks old, were obtained from the Institute of Science and Technology in Biomodels (ICTB/Fiocruz). They were kept in rooms with a controlled environment (22°C ± 2°C) with a 12-hour light-dark cycle (light from 7 am to 7 pm). Food and water were available *ad libitum*. The project was approved by the Institutional Animal Experimentation Ethics Committee (CEUA/IOC-030/2019).

### Pharmacokinetics and biodistribution study

Swiss Webster mice were administered 10 mg/kg (primaquine base) of either: (i) primaquine diphosphate (Sigma-Aldrich) solution in water (“Pq,” *n* = 7 mice per timepoint); (ii) primaquine-loaded galactose-containing nanoemulsion (“NE-Pq,” *n* = 5 mice per timepoint); or (iii) primaquine-loaded galactose-containing nanochitosan (“NQ-Pq,” *n* = 7 mice per timepoint). All formulations were given by gavage with a final volume of 200 µL. At different intervals (0.5, 1, 2, 4, 8, and 24 hours), animals were subjected to anesthesia with a mixture of xylazine (10 mg/kg) and ketamine (100 mg/kg). Blood was collected by cardiac puncture, plasma was separated by centrifugation, RBCs were washed three times in saline, and plasma and RBC were stored at −20°C. After the animal was exsanguinated, the liver, lungs, and spleen were collected and frozen in liquid nitrogen until processed for primaquine quantification.

### Sample preparation

The extraction procedure was based on Carmo et al. (22) and Joshi and Devarajan (23). Briefly, 100 µL or 100 mg of the sample was transferred to a 1.5 mL microtube. Then, 100 µL of 2% acetic acid-acidified acetonitrile and 100 µL of ultrapure water were added. Next, the samples were homogenized for 1 minute using a vortex, and 50 µL of 12.5% zinc sulfate solution was added. The samples were homogenized again for 1 minute and left to stand for 20 minutes to ensure complete protein precipitation. After this period, the samples were centrifuged at 7,500 rpm for 15 minutes at room temperature. Finally, the supernatant was collected, and 20 µL was injected into the liquid chromatograph. This process was repeated to construct the eight-point calibration curves (0.39–50 µg/mL).

### Analytical procedures

The primaquine concentrations in plasma, liver, lung, and spleen samples from mice treated with free primaquine were determined using high-performance liquid chromatography with a UV detector (HPLC-UV). Method validation followed orientations of the Guidance for Bioanalytical Method Validation from the Food and Drug Administration. The method was developed using a Waters liquid chromatography system with an Alliance e2695 Separation Module, quaternary pump, autosampler, degasser, column heater, and a dual channel UV–Vis detector (Milford, USA). The Empower 3 software was used for system control, peak integration, and data analysis. The separation was performed using an XBridge C18 reverse-phase column (150 × 4.6 mm, 5 µm) and an XBridge C18 reverse-phase pre-column (20 × 4.6 mm, 5 µm). For elution and identification of the primaquine, the mobile phase (MP) consisted of (A) potassium phosphate buffer (0.02 mol/L, pH = 3) and (Bi) acetonitrile. A simple flow gradient was adopted to accelerate primaquine elution (90% A and 10% B up to 1.9 min; 75% A and 25% B in the range of 1.9–8.0; 90% A and 10% B up to 13 min; Table S1 ). The flow rate of the MP was set at 1.0 mL/min, the column oven temperature was maintained at 35°C, and the UV detector was set at 266 nm. An injection volume of 20 µL was used, and the total run time was 13 minutes.

### Data analyses

The physicochemical analyses of the nanoparticles were performed in triplicate and are expressed as the mean value ± SD. Statistical significance tests were applied through mixed-model analysis of variance, using Prism GraphPad Software. The results of the HPLC analysis for each time point of plasma or tissue samples were used to plot drug concentration vs time, generating decay curves for the Pq, NE-Pq, and NQ-Pq analytes. Non-compartmental pharmacokinetic analysis was conducted using PK Solutions 2.0 (Summit Research Services, Ashland, USA). The area under the concentration-time curve was calculated using the trapezoidal method (AUC0-t) from time zero to the last data point and extrapolated to infinity (AUC0-∞). The maximum plasma concentration (Cmax) and the time taken to reach Cmax (Tmax) were obtained from the experimental data. The elimination rate constant (K) was determined from the slope of the regression line, allowing the calculation of the elimination half-life (t1/2β) using the formula 0.693 /K (24). All data were presented as mean ± SD.

## RESULTS

### Nanostructure generation and physicochemical properties

**Fig 1:**
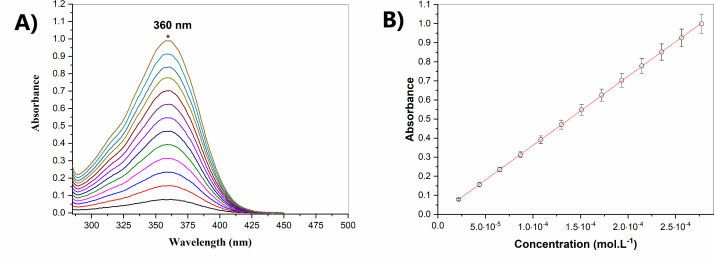
Absorption spectra of primaquine. (**A**) Absorption profile of primaquine diphosphate solution at the concentration range 21.8–277.3 µM in methanol as solvent was used to quantify and analyze the activity after incorporation in the nanoemulsion (NE-Pq) and nanochitosan (NQ-Pq) formulations containing D-galactose by absorption spectrum measurement in the UV-Vis region (230–500 nm). (**B**) Line equation *y* = 3621.722*x* – 6.67 × 10^–4^ and *r*^2^ = 0.99998.

**Fig 2:**
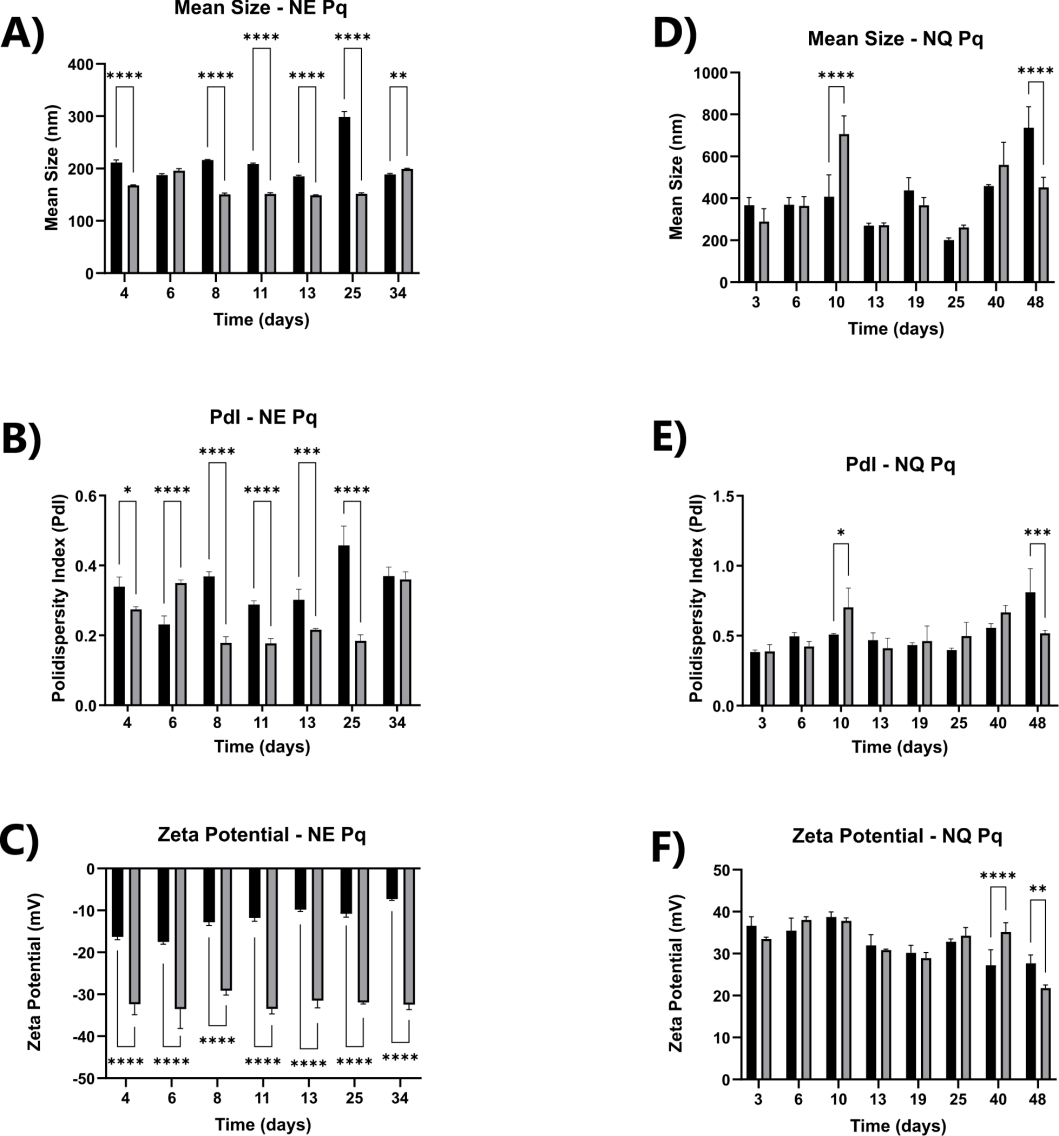
Physicochemical characterization of primaquine nanoemulsion (NE-Pq) and primaquine nanochitosan (NQ-Pq) formulations. The black bars represent the loaded (primaquine- and galactose-containing) nanoparticles, and the gray bars represent the empty (no primaquine and no galactose) nanoparticles. (**A–C**) Mean size (**A**), PDI (**B**), and zeta potential (**C**) of the NE-Pq formulation, showing that this nanoformulation presented size around 200 nm, PDI lower than 0.3, and zeta potential greater than −20 over 30 days. (**D–F**) Mean size (**D**), PDI (**E**), and zeta potential (**F**) of the NQ-Pq formulation, showing that this nanoformulation presented an average size of around 400 nm, PDI of 0.5, and zeta potential around +30 over 48 days. Empty particles containing neither primaquine nor galactose were analyzed in parallel.

The absorption spectra of primaquine diphosphate in methanol analyzed under the UV-Vis region (230–500 nm) showed an absorption peak at 360 nm, proportional to the concentration range from 21.8 to 277.2 µM, shown in Fig. 1A. As observed, the linear relation within this concentration range is close to the unit (Fig. 1B). The nanoemulsions (NE-Pq) prepared with primaquine diphosphate and D-galactose in concentrations of 2.15 mg/mL of each chemical showed an average size distribution around 200 nm with one stable nanoformulation PDI lower than 0.3 over 30 days of shelf bench, therefore, leading to stability in the analyzed period (Fig. 2A through C). An average of 45% of the incorporation of the activity was observed in the nanostructured system. For the chitosan nanoparticles (NQ-Pq) with primaquine diphosphate and D-galactose at the final concentration of 2.22 mg/mL, the nanoformulation had an average size of around 400 nm and a PDI of 0.5 and a shelf bench period over 48 days (Fig. 2D through F) and an average of 50% of incorporation of the activity. For the NQ-Pq formulation, some fluctuations in the above parameters were observed over time. Significant differences in particle size were detected on days 13 and 25, as well as some fluctuations in PDI and zeta potential were observed. However, these fluctuations did not seem to impact on the general stability of the preparations, as evidenced by characteristics such as non-aggregation and physical structure. Therefore, the fluctuation in size seemed to be punctual, and the particles were stable for up to 30 days. The results allowed us to conclude that the nanoformulations’ size and stability fit most of the requirements for biological and *in vivo* application.

### Pharmacokinetics

**Fig 3:**
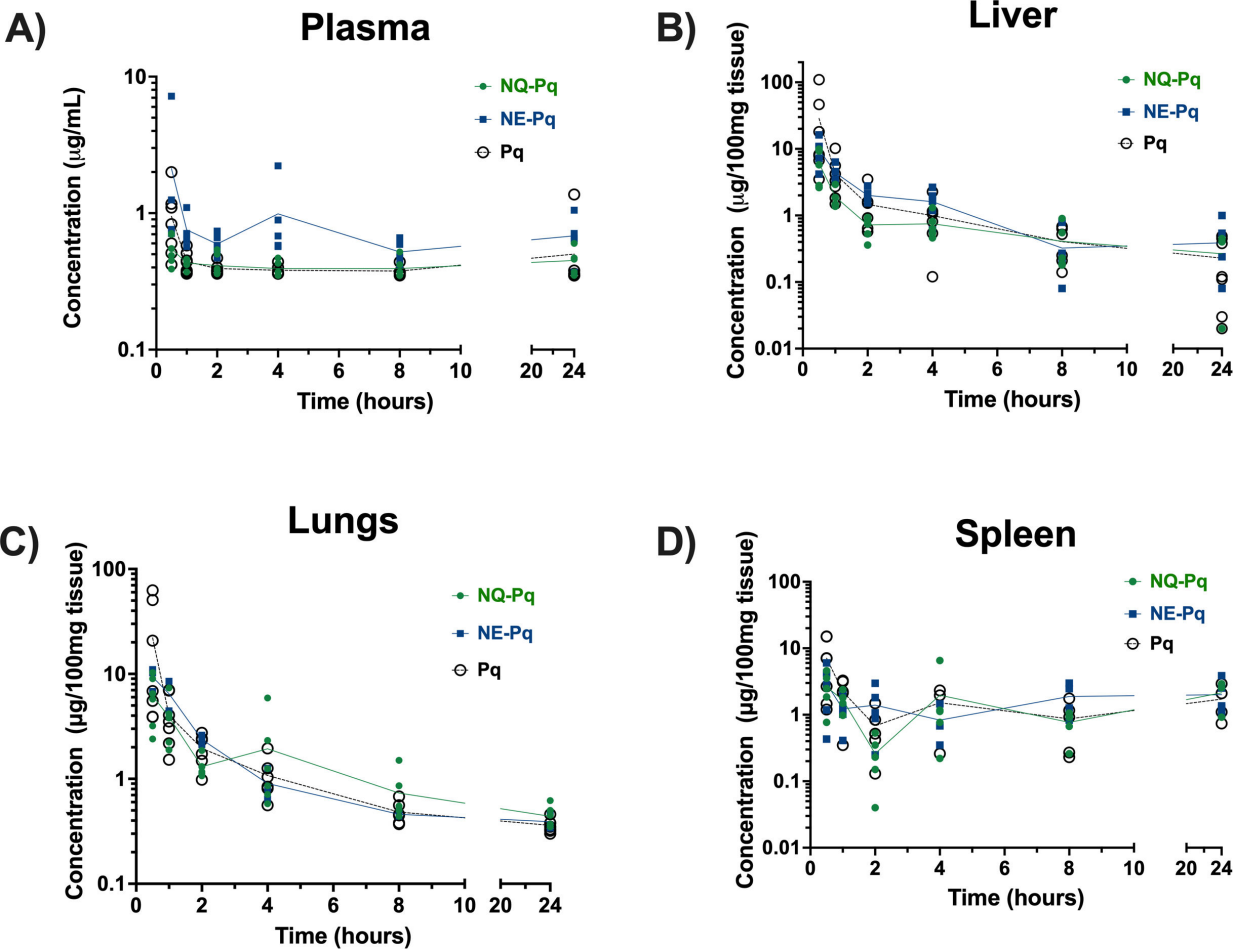
Pharmacokinetics of primaquine delivered as free drug (Pq), nanoemulsion (NE-Pq), or nanochitosan (NQ-Pq) formulations in plasma and organs. Primaquine diphosphate was prepared as free primaquine (Pq), primaquine nanoemulsion (NE-Pq), or primaquine nanochitosan (NQ-Pq) and given to Swiss Webster mice (all at 10 mg/kg free base) by gavage. Mice (*n* = 5–7) were killed at time intervals of 0.5, 1, 2, 4, 8, and 24 hours after drug administration, the plasma, liver, lungs, and spleen were harvested, and primaquine concentrations were analyzed by HPLC.

Oral administration of free primaquine diphosphate in an aqueous solution resulted in peak concentrations at 30 minutes in plasma, and the liver, lungs, and spleen, followed by decay at 1–2 hours and a relative plateau in the next 24 hours (Fig. 3A through D). Primaquine concentrations in plasma varied between 0.4 and 2.0 µg/mL among animals at 30 minutes (Fig. 3A). A broader variation was observed in the organs. In the liver, primaquine concentrations varied between 3.5 and 110 µg of drug per 100 mg of tissue at 30 minutes (Fig. 3B), with a similar quantitative profile in the lungs and spleen (Fig. 3C and D). Primaquine concentrations in RBC, for all formulations, were below the limit of quantification of the method (data not shown).

The pharmacokinetic parameters showed revealing data. In the liver, the Cmax of primaquine resulting from the control formulation (Pq) was about twice as much as the Cmax observed with the galactose-based nanoformulations (Table 1). On the other hand, the half-life and the mean residence time (MRT) of primaquine in the liver were three times higher with the nanochitosan-galactose formulation than with free primaquine, and the volume distribution was four times higher. In the lungs, the nanoemulsion-galactose formulation showed half-life, MRT, and volume distribution two times greater than the free primaquine formulation (Table 1).

**TABLE 1:** Pharmacokinetic parameters after intragastric administration of free primaquine (Pq), primaquine-nanoemulsion (NE-Pq), or primaquine-nanochitosan (NQ-Pq) formulations in mice

	Cmax (mg/mL)	Half-life (hour)	MRT (hr)	AUC(0 t) (mg-hour/mL)	AUC¥ (mg-hour/mL)	Vd (area) (mL)	CL (area) (mL/hour)
*Plasma*							
Pq	0.9	104.1	150.2	9.1	62.7	718.0	4.8
NE-Pq	2.1	23.9	34.5	15.2	38.7	266.9	7.7
NQ-Pq	0.6	67.2	96.9	10.1	53.7	541.5	5.6
*Liver*							
Pq	13.4	3.5	5.0	19.0	20.0	74.9	15.0
NE-Pq	7.9	2.4	3.4	17.8	18.9	54.7	15.9
NQ-Pq	5.7	10.5	15.1	11.3	14.4	315.6	20.9
*Lungs*							
Pq	14.3	3.0	4.3	23.2	24.7	52.3	12.2
NE-Pq	5.5	7.0	10.1	21.2	25.4	119.5	11.8
NQ-Pq	8.5	2.9	4.2	19.0	20.5	61.1	14.6

In the plasma, the effect was the opposite. Both nanoformulations showed shorter half-lives, MRT, and smaller distribution volumes than the free primaquine formulation (Table 1). The nanoemulsion formulation generated pharmacokinetic values (half-life, MRT, and Vd) 63%–77% lower and the nanochitosan formulation 25%–36% lower than the free primaquine solution.

These effects meant that in mice receiving free primaquine solution, the liver/plasma ratio for the volume of distribution was 0.1. In contrast, in mice receiving the nanochitosan, this ratio increased to 0.58, nearly six times higher. Similarly, the liver/plasma ratios for primaquine half-life and mean resident time were almost five times higher in mice receiving the nanochitosan vs the free primaquine formulations. These numbers evidence an unequivocally improved performance and higher bioavailability of the nanochitosan formulation regarding liver targeting.

## DISCUSSION

The present study shows that a galactose-containing primaquine-loaded nanochitosan formulation induced a more favorable profile of primaquine distribution in the liver than a free primaquine solution and the other nanoemulsion drug delivery system evaluated. This good profile was characterized by substantially extended half-lives and MRT of primaquine in the liver, with a marked increase in the volume of distribution and, conversely, shorter half-life and MRT and smaller volume of distribution in the plasma.

New drug delivery systems based on natural or synthetic materials have been exponentially developed and used successfully in the last decade. These include nanoparticles (25), nanoemulsions (26, 27), liposomes (28, 29), and polymeric mix systems (30, 31). Nanomaterials are used as delivery vehicles for different therapeutic compounds. Their small size (100–500 nm), biocompatibility, and sustained drug release (32) permit delivery to a specific biological site, leading to greater therapeutic efficacy. Nanocarriers enhance drug pharmacokinetics and pharmacodynamics and improve cellular, organ, tissue, and molecular targeting (33). Nanoemulsions are nano-sized emulsions manufactured to enhance the delivery of active pharmaceutical ingredients. These are thermodynamically stable isotropic systems in which two immiscible liquids are mixed to form a single phase using an emulsifying agent, i.e., surfactant and co-surfactant. The droplet size of nanoemulsion falls typically in the 20–200 nm range. Also, nanochitosan, a natural polymer obtained by alkaline hydrolysis of chitin, consisting of randomly distributed β-(1, 4)-linked D-glucosamine (deacetylated) and N-acetyl-D-glucosamine (acetylated units), shows excellent physicochemical properties, is environmentally friendly and bioactive, and can be used as a vehicle for incorporating a wide range of compounds including antimicrobial agents or antioxidants (34, 35). The nanoparticles can be mixed with ligands that can help to deliver a compound to a specific target organ. Nanoformulations have indeed been considered in antimalarial therapy to overcome limitations of currently available drugs, for instance, low bioavailability, different pharmacokinetics in the case of combination therapies, and toxicity (36). In the present study, the primaquine nanochitosan formulation showed relatively larger sizes, around 400 nm, as observed in previous studies (37), and some fluctuation over time in the physicochemical parameters, including particle size, polydispersity index, and zeta potential. However, these fluctuations did not seem to impact the general stability of the preparations, as evidenced by characteristics such as non-aggregation and physical structure. Administration of the nanochitosan formulation led to improved delivery of primaquine to the liver; therefore, further development of this formulation is warranted. Process optimization should improve the level of the drug encapsulation activity, establish the shelf lifetime, and define the scaling process for future applications.

Primaquine use is intended to destroy *P. vivax* hypnozoites, which reside in hepatocytes; therefore, drug accumulation in the liver is desired. On the other hand, in patients with G6PD deficiency, cumulative levels of primaquine in the blood may lead to oxidative stress in vulnerable RBCs, which, depending on the intensity, can cause massive hemolysis, hemoglobinuric kidney failure, and death (38). Therefore, formulations that promote extended residence and greater volume of distribution of primaquine in the liver and, conversely, away from the blood should be considered ideal for best efficacy and decreased hemotoxic potential. This desired combination of pharmacokinetic features was achieved in this study with the nanochitosan-galactose formulation, generating prospects for further evaluation of its potential as a more productive and safer alternative for *P. vivax* radical cure. The improved delivery of primaquine to the liver by the nanochitosan formulation may be related to factors such as its mucoadhesive properties (39) and its cationic surface charges (compared to the negatively charged nanoemulsion formulation), which may help with cellular uptake (40, 41). Curiously, the mean size of the nanochitosan particles was close to 400 nm, which is larger than the average liver sinusoid fenestrae diameters (50–300 nm) (42). However, previous studies have shown primaquine-loaded chitosan nanoparticles with sizes in the range of 287–686 nm (43), and liposomes sized 200 and 400 nm were shown to be heavily uptake by liver cells, both hepatocytes and Kupffer cells (44). Mechanisms could include the potential deformability of the nanoparticles, allowing penetration through the pores, or the particles could be transported by liver sinusoid endothelial cells by transcytosis (45). Additional studies are necessary to determine the specific cell target of the NQ-Pq formulation. A recent study with primaquine-loaded chitosan nanoparticles obtained particles of much smaller size (47 nm), and oral administration to rats over 5 days also resulted in lower plasma Cmax but increased concentrations of primaquine in the liver (46).

The nanoemulsion formulation, on the other hand, showed no improved performance in terms of liver delivery compared to free primaquine; however, it showed a much shorter plasma half-life and MRT and a much smaller plasma volume of distribution, which indicates that higher primaquine doses with this formulation could be less toxic for RBCs.

The present study’s findings corroborate previous attempts to improve liver delivery of primaquine by targeting asialoglycoprotein receptors. Early studies in the 1980s had already indicated that this was a promising strategy, with liposomal formulations leading to increased primaquine accumulation in the liver and decreased lethality in mice (16, 17). Later, studies of primaquine formulated as chylomicron and other nanoemulsions led to similar outcomes (18). A galactose-coated dendrimer primaquine formulation increased drug circulation and liver accumulation (19). Also, a nanochitosan formulation (without galactose incorporation) of primaquine given intravenously led to a lower lethal dose than free primaquine solution (43). Tomiya and coworkers (20) showed that a poly-γ-glutamic acid modified with a synthetic TriGalNAc resulted in marked accumulation in the liver of mice after intravenous injection and a synthetic conjugate containing primaquine resulted in increased uptake by rat hepatocytes. Kumar et al. (47) analyzed the performance of a primaquine-loaded galactosylated gelatin nanoparticle formulation, which induced less hemolysis *in vitro* than free primaquine (although the level of hemolysis even for free primaquine was low overall). Pharmacokinetic and biodistribution evaluations showed increased accumulation of primaquine in the liver after 7-day intraperitoneal treatment of Sprague-Dawley rats with the nanoparticle formulation compared to free primaquine. The nanoparticle also showed an increased half-life of primaquine in the plasma, which was regarded as an advantage but could also be considered a downside, as it potentially increases the exposure of RBCs to primaquine. The use of primaquine-containing formulations with dextran sulfate as a carbohydrate polymer (“nanocarboplex”) with pullulan acting as asialoglycoprotein receptor ligands resulted in no difference in plasma pharmacokinetics but a higher accumulation of primaquine in the liver after 1 and 4 hours following intravenous administration, compared to free primaquine (23). In the absence of pullulan, the nanocarboplex formulation’s liver-targeting was ineffective.

In most of these studies, the primaquine nanoparticle formulations were delivered intravenously, representing a disadvantage regarding the real-life treatment of patients in the field. Our nanochitosan and nanoemulsion formulations were given orally and achieved improved pharmacokinetic performances compared to oral-free primaquine.

Pharmacokinetic and pharmacodynamic data with primaquine obtained in the mouse have limitations and cannot be directly translated to humans due to distinct species characteristics but also to the differences in the weight-based dose and use of single-dose administration in uninfected animals. In rodents, higher doses compared to those given to humans are commonly used for more consistent detection of the drug in the plasma and selected organs at various timepoints. For instance, Fasinu and coworkers used a 45 mg/kg dose of primaquine for comparative pharmacokinetics of R and S primaquine enantiomers in mice (48). In the present study, a dose equivalent to 10 mg/kg of primaquine base was used, whereas the usual clinical dose of primaquine varies between 0.10 and 0.75 mg/kg. As a result, the single dose of free primaquine resulted in Cmax plasma concentrations of 0.4–2.0 μg/mL, which are expectedly higher than those reported in the literature for the human treatment, with Cmax around 0.1 µg/mL in plasma (49–51). However, despite these limitations, pharmacokinetics and metabolism of primaquine enantiomers have been shown to be similar in human volunteers and mice receiving a single oral dose (48, 51). That was also true for the antimalarial amodiaquine, and in that case, drug exposure was predicted to be higher in humans than in mice (52). Our results show that, within the same biological system (the mouse), primaquine delivered through a nanochitosan formulation had improved pharmacokinetic characteristics compared to free primaquine. Therefore, it is reasonable to expect that similar outcomes would be achieved in humans.

Different drug delivery strategies have indeed been considered in antimalarial therapy to overcome limitations of currently available drugs, for instance, low bioavailability, different pharmacokinetics in the case of combination therapies, and toxicity (12, 53–55). However, when considering the potential of these novel primaquine formulations for treating *P. vivax* relapses in patients in the field, there could be limitations that extrapolate their intrinsic advantages, such as stability of the formulations, ease of storage, distribution, administration, and cost. In this study, in lab conditions, both nanoformulations showed good stability at room temperature for several weeks, presenting average size, polydispersity index, and zeta potential as expected. The shelf lifetime studies also indicate that the formulation is well stable with good conditions for the scaling-up process. However, additional evaluations of extended periods and storage conditions, including higher temperatures mimicking field conditions, are necessary.

Regarding cost and commercial potential, many nanopharmaceuticals and nanomedicines are currently commercially available in the market, showing that these formulations are competitive (56, 57). In addition, formulations in which the active drug can be used at lower doses and result in higher efficacy and a more favorable safety profile (resulting in less hospitalization, for instance) could have economic advantages. Finally, primaquine use is restricted in many places due to concerns regarding hemolysis, or a G6PD activity test is required before treatment, increasing costs and/or severely limiting its use. Therefore, a targeted, safer formulation, developed with accessible raw material and a lower final cost of scaling up the process, could make sense economically.

The data shown here are auspicious for the potential of these two nanostructured primaquine formulations, particularly the nanochitosan formulation. However, additional studies to determine the effects of repeated dosing on liver and plasma levels, prevention of hemolysis upon treatment of G6PD-deficient animals, and efficacy of different doses against the liver stages, especially the hypnozoites, are necessary to confirm this potential and stimulate further evaluations, including clinical trials. And whether these formulations can also improve the pharmacokinetics of the newly available anti-hypnozoite drug tafenoquine is worthy investigating.

## SUPPLEMENTAL MATERIAL

Supplemental material aac.00915-23-s0001.doc Scheme of nanostructure preparation and parameters for HPLC gradient elution.

ASM does not own the copyrights to Supplemental Material that may be linked to, or accessed through, an article. The authors have granted ASM a non-exclusive, world-wide license to publish the Supplemental Material files. Please contact the corresponding author directly for reuse.
