# Supplementary material for: A nanochitosan-D-galactose formulation increases the accumulation of primaquine in the liver
Source: Antimicrob Agents Chemother. 2024 Mar 22;68(5):e00915-23. doi: 10.1128/aac.00915-23 (PMC11064505; doi:10.1128/aac.00915-23)
Supplement: Supplemental material — Scheme of nanostructure preparation and parameters for HPLC gradient elution. [file aac.00915-23-s0001.doc]

**
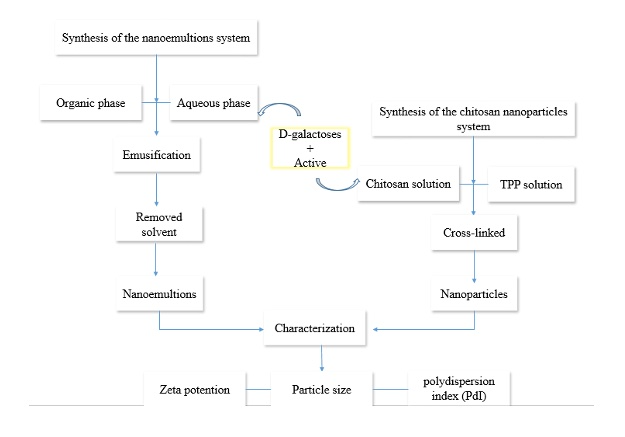
**

**Supplemental Figure 1.** Simplified scheme of the preparation of nanostructures

**Supplemental Table.** Parameters for HPLC gradient elution. A = potassium phosphate buffer (0.02 mol/L, pH = 3); B = acetonitrile.

| **Run time (min)** | **Flow (mL/min)** | **%A** | **%B** |
| --- | --- | --- | --- |
|  | 1 | 90 | 10 |
| 1.0 | 1 | 90 | 10 |
| 1.9 | 1 | 75 | 25 |
| 8.0 | 1 | 75 | 25 |
| 9.0 | 1 | 90 | 10 |
